# Fueling CARs: metabolic strategies to enhance CAR T-cell therapy

**DOI:** 10.1186/s40164-024-00535-1

**Published:** 2024-07-10

**Authors:** Arne Van der Vreken, Karin Vanderkerken, Elke De Bruyne, Kim De Veirman, Karine Breckpot, Eline Menu

**Affiliations:** 1 Translational Oncology Research Center, Team Hematology and Immunology, Vrije Universiteit Brussel, Laarbeeklaan 103, Brussels, 1090 Belgium; 2 Translational Oncology Research Center, Team Laboratory of Cellular and Molecular Therapy, Vrije Universiteit Brussel, Laarbeeklaan 103, Brussels, 1090 Belgium

**Keywords:** CAR T cells, Co-stimulus, Drug repurposing, Metabolism, Mitochondria

## Abstract

CAR T cells are widely applied for relapsed hematological cancer patients. With six approved cell therapies, for Multiple Myeloma and other B-cell malignancies, new insights emerge. Profound evidence shows that patients who fail CAR T-cell therapy have, aside from antigen escape, a more glycolytic and weakened metabolism in their CAR T cells, accompanied by a short lifespan. Recent advances show that CAR T cells can be metabolically engineered towards oxidative phosphorylation, which increases their longevity via epigenetic and phenotypical changes. In this review we elucidate various strategies to rewire their metabolism, including the design of the CAR construct, co-stimulus choice, genetic modifications of metabolic genes, and pharmacological interventions. We discuss their potential to enhance CAR T-cell functioning and persistence through memory imprinting, thereby improving outcomes. Furthermore, we link the pharmacological treatments with their anti-cancer properties in hematological malignancies to ultimately suggest novel combination strategies.

## Background

Recently, Dr. Carl June of the University of Pennsylvania received the prestigious 2024 Breakthrough Prize in Life Sciences, one of the largest science awards globally, for his groundbreaking contributions to the development of chimeric antigen receptor (CAR) T-cell therapy. This recognition underscores the profound significance of CAR T-cell therapies in the landscape of modern medicine. CAR T-cell therapy represents a revolutionary approach to treat cancer, which is based on killing cancer cells in an HLA-independent context by infusing the patient with their own T cells. These T cells are administered after they have been engineered ex vivo to improve their natural ability to effectively kill target cells. The versatility of CAR T-cell therapy extends beyond hematological malignancies, with ongoing research exploring its application in treating solid tumors, advancing HIV research, fibrosis, and managing autoimmune diseases [1–4].

Currently, six CAR T-cell products are FDA approved. Four of them (tisagenlecleucel, axicabtagene ciloleucel, lisocabtagene maraleucel and brexucabtagene autoleucel) target CD19 for the treatment of relapsed and/or refractory (R/R) B-cell lymphomas such as diffuse large B-cell lymphoma (DLBCL), and B-cell lymphoblastic leukemia (B-ALL) [5–10]. Ciltacabtagene autoleucel (cilta-cel) and idecabtagene vicleucel (ide-cel) are currently the two FDA approved CAR T-cell products targeting B-cell maturation antigen (BCMA) for the treatment of R/R Multiple Myeloma (MM) [11, 12]. These therapies are approved for relapsed patients in ≥ 2nd line for B-cell lymphomas, leukemias and Multiple Myeloma. Despite overall good response rates, a majority of MM patients and a significant number of patients with B-cell lymphomas eventually relapse [13]. One major reason for therapy failure includes the loss of CD19 or BCMA, and a Darwinian selection of antigen negative clones [14, 15]. For instance, 4–33% of MM patients treated with BCMA-directed CAR T cells, suffer from BCMA loss [11, 16, 17]. The most common reason for antigen-positive failure is the lack of T-cell persistence and exhaustion [18]. A total of 85% of B-ALL patients treated with CD19 CAR T cells showed initially remission, however almost half of them eventually relapsed, with no detectable CAR T cells in their blood [19]. Hence, durable and fit CAR T cells are needed for a good response.

After apheresis, T cells are genetically modified and reinfused into a patient. Hence, each patient receives another composition of T-cell subsets. Studies have shown that patients with poor responses to CAR T-cell treatment have higher levels of PD-1 and LAG-3 after apheresis, suggesting a lower overall T-cell fitness [20]. The transcriptional program of responding patient also differs from patients with partial response or progressive disease. CAR T cells of non-responders have been described as effector T cells, with an exhaustion, glycolytic and apoptotic gene signature, while patients who achieved complete responses have a manyfold higher frequency of CD8^+^ CAR T cells with a memory phenotype and gene signature [21–25]. Moreover, the percentage of memory subsets in the infused CAR T-cell product is associated to better clinical responses since they have a higher proliferative capacity [21–23, 25, 26]. Memory T cells possess a less differentiated phenotype and are essential for a durable anti-tumor effect due to their superior expansion potential, long-term persistence, and ability to become effectors upon encountering antigens. Hence the long-term persistence is a feature of a less differentiated memory status [27–29]. Another important point to stress out is that MM is predominantly a malignancy of elderly. Aged T cells are associated with a range of molecular changes, including mitochondrial dysfunction, alongside genetic and epigenetic alterations. This results in senescent T cells and an imbalance of naïve-memory-effector T cells , which is also the case in MM patients [30–32]. Therefore, autologous T cells, collected via apheresis and used for CAR T-cell production, could lead to an already pre-dysfunctional product.

It has become clear that modulation of (CAR) T-cell metabolism leads to an improvement of therapy effectiveness and outcome. In this review, we delve into the metabolic profiles of the different T-cell subsets, how CAR design shapes T-cell metabolism and fate, and discuss metabolic strategies – be they genetic interventions or pharmacological strategies – to improve therapy. For the latter, we also discuss the anti-cancer properties of these drugs in hematological malignancies, which may potentially exhibit additive or synergistic effects in combination with CAR T cells.

## Metabolism and T-cell fate are inextricably linked

T cells comprise a heterogeneous pool of cells with several differentiation states. Different T-cell subsets are known including naïve T cells (CD25^−^,CD95^−^), effector T cells (CCR7^−^,CD62L^−^, CD45RA^+^, CD45RO^−^) and memory T cells, which can be further subdivided in stem cell memory T cells (Tscm, CD95^+^, CCR7^+^, CD62L^+^, CD45RA^+^, CD45RO^−^), central memory T cells (Tcm, CCR7^+^, CD62L^+^, CD45RA^−^, CD45RO^+^) and effector memory T cells (Tem, CCR7^−^, CD62L^−^, CD45RA^−^, CD45RO^+^). The memory-related phenotypic markers CD62L (also known as L-selectin) and CCR7 play a crucial role in T-cell homing and trafficking in lymph nodes [33, 34]. It is important to note that T-cell differentiation is not a linear process, and precursor-exhausted T cells can originate from diverse subsets before ultimately differentiating to terminally exhausted T cells [35, 36].

The metabolism of all these subsets is highly different, depending on their differentiation status and energy demands with effector T cells having increased glycolysis and memory T cells relying more on mitochondrial oxidation of nutrients. Glucose and glutamine catabolism is upregulated by mammalian target of rapamycin (mTOR) signaling to produce rapid and sufficient ATP, while mitochondrial oxidation is regulated by AMP-activated protein kinase (AMPK) [37–39]. The metabolism of T cells is linked to their lifespan. Highly glycolytic cells are found to have a short lifespan, whereas long-living proliferating T cells are directly associated with mitochondrial oxidative phosphorylation (OXPHOS) [40]. Inhibition of OXPHOS is sufficient to induce an exhaustion related gene signature, ending in apoptosis [41]. Here we discuss in detail how signal transduction and metabolism relate to T-cell fate.

**Fig. 1:**
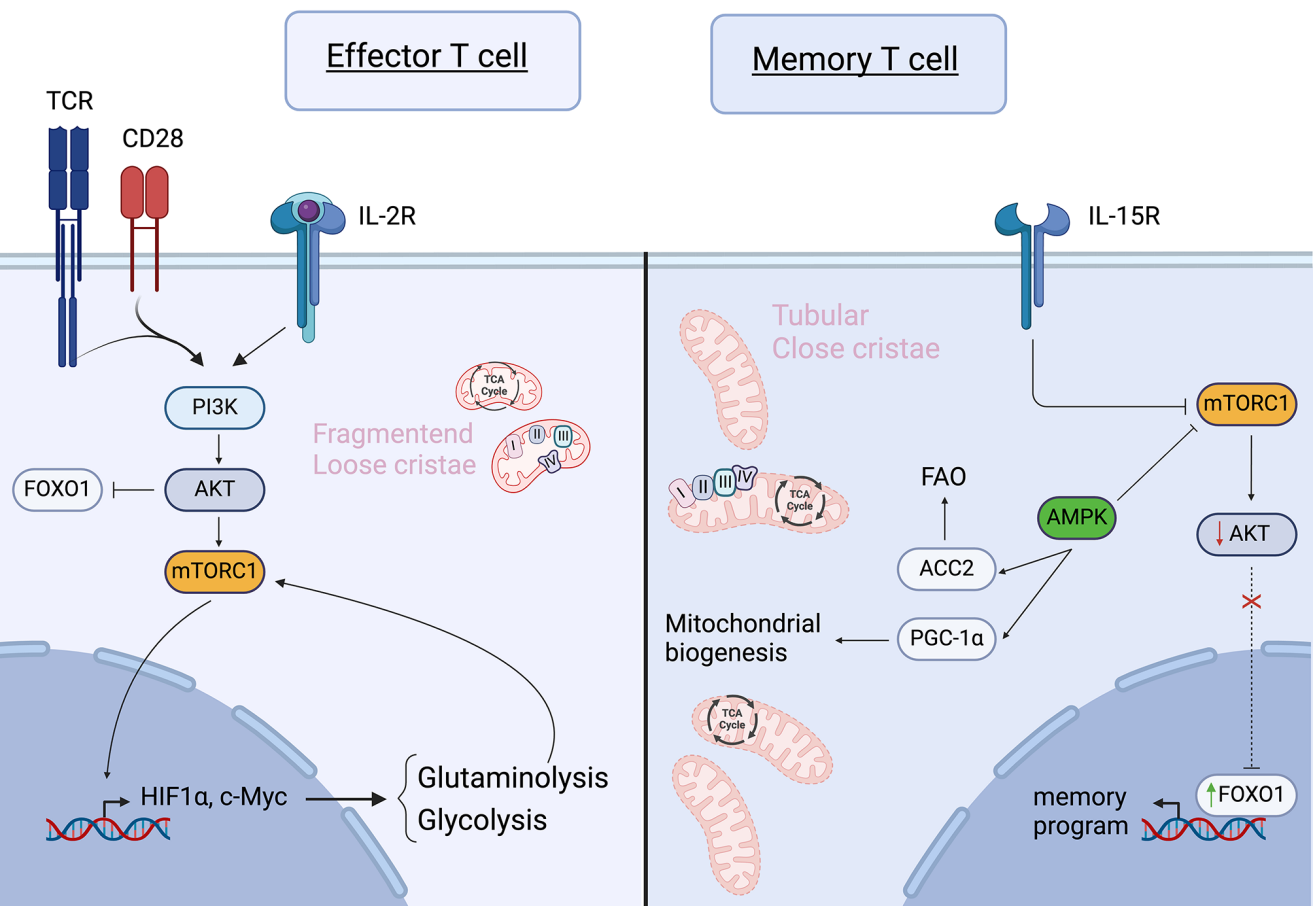
Signal transduction and its relation to metabolism in effector and memory T cells. Optimal effector T-cell signaling is induced by a combination of signals from an activated T-cell receptor (TCR), CD28 co-stimulation, and cytokines such as IL-2. Together, these signals activate the PI3K/Akt/mTORC1 pathway, leading to the activation of glycolytic genes. Activated Akt inhibits FOXO1. In memory T cells, mTORC1 signaling is downregulated by AMPK and cues such as IL-15R signaling. The reduced mTORC1/Akt activity results in the activation of the transcription factor FOXO1, which induces a memory gene signature. Memory T cells have an increased mitochondrial mass with more tubular cristae, facilitating close proximity between the different complexes of the electron transport chain. In contrast, effector T cells exhibit mitochondria with loose cristae and increased physical distance between the complexes of the electron transport chain. (PI3K: Phosphatidylinositol 3-kinases, PGC-1α: Peroxisome proliferator-activated receptor-gamma coactivator 1alpha, mTORC1: mammalian target of rapamycin complex 1, HIF1α: hypoxia-inducible factor 1-alpha, ACC2: acetyl-coenzyme A (CoA) carboxylase A, AMPK: adenosine monophosphate-activated protein kinase, FAO: fatty acid oxidation, TCR: T-cell receptor, IL-2R: interleukin-2 receptor, IL-15R: interleukin-15 receptor, FOXO1: Forkhead box protein O1, Akt: protein Kinase B). Created with Biorender.com

Naïve T cells are encouraged to survive via cytokines such as IL-7. IL-7 stimulates Akt signaling, which in turn sustains GLUT1 expression for glucose uptake. Glucose is subsequently metabolized to pyruvate and transported into the mitochondria to be used either in the tricarboxylic acid (TCA) cycle or for the synthesis of triacyl glycerol (TAG), which can be used later for fatty acid oxidation (FAO). Hence, naïve T cells depend mainly on OXPHOS and FAO to meet their energy demands [42]. Upon stimulation of the T-cell receptor (TCR) and CD28, the PI3K-Akt-mTOR pathway becomes activated, promoting glycolysis through upregulation of c-Myc and HIF-1α (Fig. 1) [43]. In addition, glutamine uptake is upregulated upon T-cell activation to elevate ATP production, via mTORC1 [43, 44]. The breakdown of glutamine, glutaminolysis, further positively regulates mTORC1 [44, 45]. This metabolic shift allows T cells to differentiate from naïve T cells to effector T cells, supporting proliferation and cytokine production by generating sufficient ATP. The activation of mTORC1 drives aerobic glycolysis in effector T cells, while inhibition of mTORC1 reduces glucose uptake and impairs effector differentiation, thereby retaining memory features [46]. Memory T cells exhibit metabolic traits resembling naïve T cells, yet they perform higher levels of OXPHOS and mitochondrial spare respiratory capacity (SRC), facilitating rapid activation upon antigen re-encounter [47]. The other mTOR complex, mTORC2, negatively impacts memory formation; knock-out of its subunit Rictor leads to upregulation of Eomes and TCF-1 (transcription factors related to memory T cells), mediated via FOXO1 stabilization in the nucleus. FoxO1 stabilization is associated with increased SRC and FAO, favoring memory differentiation [48]. Recently, FOXO1 is identified as a master regulator of memory imprinting in T cells [49, 50]. Both genetic and pharmacological inhibition of FOXO1 in CAR T cells results in a more exhausted phenotype and weakened anti-tumor responses, while overexpression of FOXO1 enhances anti-tumor immunity, increases mitochondrial mass and induces more stemness [49, 50].

AMPK is an energy sensor, responding to changes in the AMP/ATP ratio. AMPK signaling is inhibited by glutamine uptake, but promotes memory T-cell formation by phosphorylating acetyl-CoA carboxylase 2 (ACC2) and activation of the peroxisome proliferator-activated receptor gamma coactivator 1-alpha (PGC-1α) (Fig. 1) [44, 51]. Thereby AMPK enhances FAO and mitochondrial biogenesis, respectively [51]. It is essential to keep in mind that PGC-1α is a crucial modulator in mitochondrial biogenesis. PGC-1α activates the NRF1/2-TFAM axis to stimulate mitochondrial DNA replication and transcription, thereby increasing mitochondrial content [52–55]. Not only AMPK, but also the PI3K/Akt/mTOR-axis contributes to the regulation of PGC-1α [56]. Studies have shown that exhausted T cells experience progressive loss of PGC-1α due to PD-1-mediated Akt signaling, while overexpression of PGC-1α enhances mitochondrial activity, persistence, memory formation; resulting in improved in vivo efficacy [54, 55, 57].

It is evident that energy metabolism and mitochondria play a pivotal role during T-cell differentiation. Mitochondria undergo significant adaptions during the transition from naïve T cells to a memory and effector state. In naïve T cells mitochondria and the endoplasmic reticulum (ER) reside within the cytosol, without close proximity to each other [41, 58]. However, in activated T cells, mitochondria and ER form physical associations with the immune synapse to sustain Ca^2+^ -dependent T-cell activation and signaling [59–62]. Not only does the localization of the mitochondria change, but their morphology also undergoes remodeling throughout the course of T-cell fate determination. Naïve T cells typically contain round mitochondria, while those of memory T cells appear longer, more tubular as a result of Opa-1-mediated fusion. Opa-1 or Optic athrophy-1 is a dynamin-related GTPase and is located on the inner mitochondrial membrane, where it stabilizes and remodels cristae. In memory T cells, complexes I-IV of the electron transport chain (ETC) are closely located to each other due to the cristae structure, promoting OXPHOS and FAO [63]. This efficient proton pumping activity of complex I-IV results in a low mitochondrial membrane potential (ΔΨM) observed in both Tscm and Tcm. Opa-1 is found to be critical for memory function, but not for effector T cells [63]. Enhancing mitochondrial fusion in effector T cells by overexpressing Opa-1 promotes memory T-cell formation [63–65].

On the other hand, effector T cells possess punctuated mitochondria orchestrated by dynamin-related protein 1 (DRP1)-related fission. Fission induces cristae remodeling, leading to looser cristae and increased physical distance between complexes I-IV. This lowers ETC efficiency, promotes ROS formation and results in a high ΔΨM [66]. Consequently, effector T cells rely more on aerobic glycolysis [62, 63]. DRP1, located on the outer membrane of the mitochondria, is necessary for T-cell activation and the translocation of mitochondria towards the immune synapse [63, 66].

Mitochondrial dysfunction is an inherent characteristic of functional exhausted T cells, which undergo reprogramming towards glycolysis, albeit their glycolytic ability is also reduced, compared to effector T cells [67, 68]. Single-cell RNA-sequencing reveals impaired mitochondrial biogenesis, downregulated Opa-1 expression, and enrichment of oxidative stress associated genes in exhausted T cells [67]. In conclusion, each T-cell subset possesses distinct functional signaling, with a unique metabolism, actively influencing its fate.

## The metabolic-epigenetic crosstalk

Each T-cell subset has its specific gene signature, particularly in terms of exhaustion markers, and proliferation and effector-related genes. Metabolism appears to play an instructing role in altering gene activity to achieve a specific status and dictate the fate of T cells. There is evidence suggesting that epigenetic alterations might serve as a significant regulatory mechanism connecting mitochondrial activity to nuclear reprogramming [69]. Both histones and DNA can be modified to alter gene expression. Alterations are catalyzed by so called epiplayers, which require metabolic intermediates as co-factor or carbon source, thereby creating a crosstalk between epigenetics and the metabolism.

Histones are nuclear proteins that package DNA, and which are post-translationally modified by e.g. methylation or acetylation. Histone acetylation of lysine residues is catalyzed by histone acetyltransferases (HATs). Acetylation reduces the positive charge of lysine residues, resulting in more open DNA [70]. Histone deacetylases (HDACs) reverse this open state via chromatin condensation. HATs use acetyl-coenzyme A (acetyl-coA) as primary acetyl source to exert their function. Acetyl-CoA is oxidatively generated by either carboxylation of pyruvate, breakdown of long-chain fatty acids or degradation of amino acids such as glutamine. A reduction in cellular acetyl-coA levels correlates with decreased acetylation of histone H3 at lysine 9 (H3K9), leading to reduced transcription of IFN-γ [71]. On the other hand, treatment of exhausted or hyporesponsive T cells with acetate leads to histone acetylation, increased chromatin accessibility of the IFN-γ gene and increased IFN-γ production [72]. Inhibition of HDAC8 resulted in increased acetylation of H3K27 and induction of memory T cells in a hepatocellular carcinoma mouse model [73]. HDAC class III enzymes also known as sirtuins also deacetylates histones. For their function, sirtuins require the co-factor NAD^+^, which is a by-product of the ETC, via lactate fermentation and is *de novo* synthesized during tryptophan catabolism and the salvage pathway [74]. Therefore, the NAD^+^/NADH ratio can influence epigenetic alterations via sirtuins (SIRT). SIRT1 expression is downregulated in terminally differentiated CD8^+^ CD28^−^ memory T cells and its loss increases FOXO1 degradation and an increase in glycolysis [75]. Genetic perturbation of SIRT2 resulted in hyperreactive effector T cells with an increase in glycolysis and OXPHOS. The skew to effector T cells in SIRT2 knock-out T cells is at the expense of naïve and memory T cells [76].

The effect of histone methylation is context-dependent. For instance, trimethylation of histone 3 on lysine 27 (H3K27me3) is characterized by condensed chromatin and represses gene transcription. H3K4me3 on the other hand, results in an open chromatin, and is thus enriched in actively transcribed genes [70]. In T cells, the level of H3K27me3 is the highest in naïve and memory cells, compared to effector T cells [77]. A family of chromatin remodeling enzymes, namely α-ketoglutarate (α-KG)-dependent dioxygenases (α-KGDD), consume α-KG (intermediate during TCA, and formed during glutaminolysis) as co-substrate to exert their function. The family of α-KGDD enzymes include enzymes such as lysine demethylases (KDMs), ten-eleven translocation (TET) DNA cytosine-oxidizing enzymes and prolyl hydroxylases (PHDs) [78]. Increased levels of α-KG leads to histone H3K27 demethylation, which is associated with dysfunctional T cells. Succinate and fumarate, both TCA metabolites, are competitive inhibitors of α-KG and prevent α-KG to exert its function as co-substrate of α-KGDD [79]. A study shows that an increased succinate/α-KG ratio leads to increased chromatin accessibility of the regulatory elements of inflammatory genes [80]. 2-hydroxyglutarate (2-HG) is another antagonist for α-KG, which acts as a competitive inhibitor for TET enzymes. 2-HG is produced during TCR signaling and enhances memory T-cell formation by inhibition of TET2 and increased CD62L transcription [77]. The genetic loss of TET2 is reported to drive memory differentiation in CD8 + T cells and CAR T cells, while IL-12 drives effector formation via TET2-mediated DNA demethylation of the IFN-γ promotor [81–84].

Histone methyltransferases use S-adenosyl-methionine (SAM) as main source for methyl groups. SAM is produced from methionine via the one-carbon metabolism. T cells subject to impaired methionine uptake and metabolization have decreased H3K27me3, promoting a more stemness memory T-cell status, thereby preventing exhaustion [85]. Another study shows that SAM supplementation to activated T cells results in T-cell exhaustion by increased chromatin methylation [86]. Not only Histone methyltransferases but also DNA methyltransferases (DNMTs) use SAM as methyl donor for *de novo* DNA methylation. Exhausted CD19 CAR T cells from an ALL patient showed *de novo* DNA methylation, resulting in repression of memory-related genes like those encoding for TCF-1, while demethylation occurred in exhaustion-associated genes such as TOX [87]. Genetic deletion of DNMT3A or pharmacological inhibition with decitabine prevents *de novo* DNA methylation and exhaustion in CAR T cells, while promoting memory differentiation [88]. Dual inhibition of histone methyltransferase G9A and DNMTs shows slight improvements in a vaccination model [89]. However, its effect on CAR T cells and T-cell fate still needs to be studied.

Here we mainly focused on acetylation and methylation, however, succinylation and lactylation of histones also may have epigenetic effects on the memory/effector/exhaustion balance, but needs to be further studied [90, 91]. Overall, metabolites actively dictate epigenetic remodeling and thereby the T-cell fate. Not only the presence of the metabolite, but also the ratio between different metabolites is important in the complex balance between acetylation or methylation of histones and DNA.

## CAR T-cell generation alters T-cell metabolism

During the CAR T cells’ manufacturing process, peripheral blood mononuclear cells (PBMCs) are isolated through leukapheresis [92]. To facilitate viral transfer of the transgene into T cells, PBMCs are activated with anti-CD3/CD28 beads to promote proliferation, and transduction [92, 93]. Upon activation, naïve and resting T cells metabolically rewire from FAO to glycolysis [94]. This metabolic shift leads to differentiation of T cells into either highly-glucose dependent effector cells, or low-glucose dependent memory T cells. Following activation, lentiviral or retroviral gene transfer is conducted, followed by the cultivation and expansion of CAR T cells in cytokine-enriched culture medium. The selection of used cytokines impacts the differentiation into distinct phenotypical subsets. IL-2 is a lymphocyte growth factor that stimulates PI3K-Akt-mTOR signaling upon its interaction with the IL-2 receptor (IL2R), thus promoting glycolysis and effector differentiation [95]. This method is for instance used during the cilta-cell and ide-cell expansion processes [96]. However, IL-2 has been implicated in inducing exhaustion through an increased tryptophan catabolism [97]. IL-7 and IL-15 cytokines promote proliferation and differentiation into memory T cells. IL-7 enhances cell survival by upregulating GLUT1 and facilitate TAG synthesis through upregulation of glycerol channels [42, 98]. On the other hand, IL-15 inhibits mTORC1 signaling while enhancing mitochondrial fitness via enhanced SRC, biogenesis and increasing expression of carnitine palmitoyl transferase 1 A (CPT1A), thus favoring a Tscm-phenotype [99]. Lastly, the use of IL-21, particularly in combination with lactate dehydrogenase A (LDHA) inhibition, has been shown to favor memory stemness in T cells [100].

## CAR design reprograms T-cell metabolism

CAR constructs typically consist of a single chain variable fragment (scFv) as the extracellular domain, which acts as a binder to the target antigen. This scFv is fused to a hinge and transmembrane region, often derived from CD8α or CD28, along with intracellular signaling elements necessary for T-cell activation. In second-generation CARs, a co-stimulatory element, commonly derived from CD28 or 4-1BB, is included along with the CD3ζ chain for TCR signaling. CARs are designated based on the components they contain, with a typical notation including the antigen, co-stimulatory molecule, and zeta element. For example, a CAR targeting CD19 with CD28 co-stimulation and CD3ζ signaling would be denoted as CD19-CD28:ζ.

Comparison between CD19-CD28:ζ and GD2-CD28:ζ CARs reveals differences in their CD3ζ phosphorylation levels. GD2-CD28:ζ shows some phosphorylation attributed to CAR clustering, later referred to as tonic signaling [17]. In a study by Lakhani et al., the T-cell metabolism of seven different CAR T cells, distinguished solely by their scFv, was analyzed. Remarkably, even without antigen stimulation, CARs containing a rituximab-derived scfv for CD20 and 14g2a for GD2 display heightened glucose consumption and glycolysis compared to other CD20 scFv variants [101]. Hence, it seems that it is the antigen binding moiety, devoid of signaling function, that rewires T-cell metabolism, presumably via CAR clustering. To mitigate scFv-induced tonic signaling, the antigen binding moiety can be replaced by a heavy chain variable fragment, also known as nanobody, to form a nanoCAR [102, 103].

Extensive research has been conducted for the optimal co-stimulatory domain in CAR design, with CD28 and 4-1BB as the most widely utilized options. CD28 and 4-1BB command each a unique signaling pathway which consequently regulates other immunometabolic pathways to generate sufficient ATP to sustain T-cell activation and activity [104]. Stimulation of CD28 triggers the activation of the PI3K-Akt-mTOR axis, leading to a cascade of events, including the upregulation of GLUT1 expression to enhance glucose uptake. Additionally, it drives gene expression of key glycolytic enzymes such as Hexokinase II and LDHA, which play crucial roles in conserving the intracellular ATP/ADP ratio [105–107].

4-1BB (CD137 or TNFRSF9) belongs to the tumor necrosis factor receptor (TNFR) gene family and exhibits induced protein expression during T-cell priming, in addition to being present on other cell types like NK cells and dendritic cells. Within its cytoplasmic tail, interactions with TRAF-1 and TRAF-2 have been shown [108, 109]. Notably, TRAF-1 levels surge post-T-cell activation, whereas TRAF-2 is constitutively expressed in resting T cells. Upon engaging its ligand, 4-1BB employs two distinct signaling mechanisms to bolster T-cell survival. Through TRAF-2, it orchestrates NF-κB-dependent upregulation of pro-survival genes like Bcl-XL, Bcl-2, and survivin, and stimulation of p-38 MAPK [108–113]. Conversely, TRAF-1 operates in an NF-κB-independent manner, activating ERK, thereby contributing to the downregulation of the pro-apoptotic factor BIM [109]. NF-κB serves as a regulator of cellular metabolism, stimulating aerobic glycolysis and mitochondrial respiration, while p-38/MAPK upregulates PGC-1α [113]. Consequently, co-stimulatory signals mediated via NF-κB and p38/MAPK tend to rely more heavily on mitochondrial metabolism for sustaining cellular functions.

**Fig. 2:**
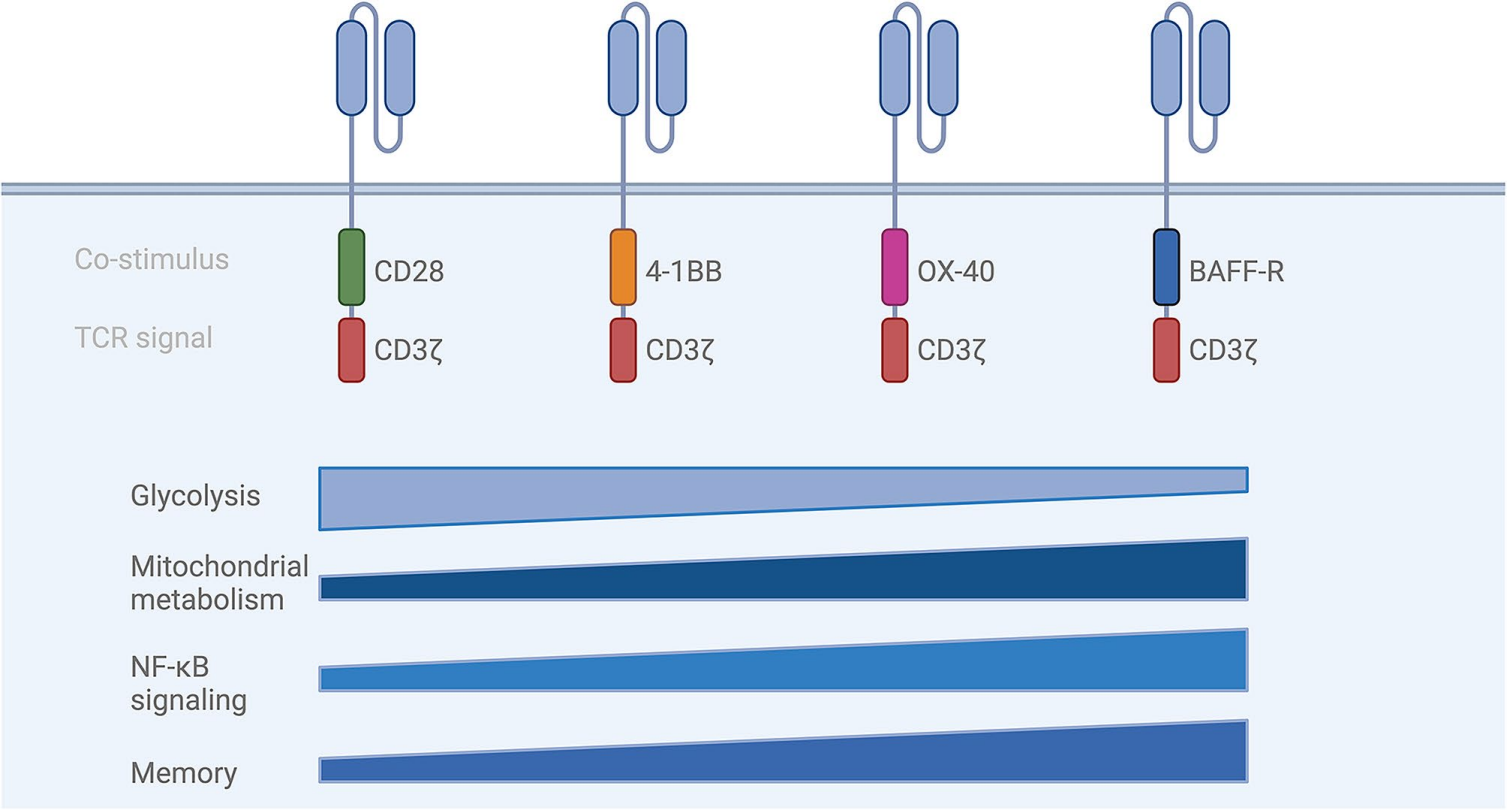
(Metabolic) effects of different co-stimuli. Each co-stimulatory domain in CAR T cells engages unique immunometabolic signaling pathways, leading to distinct phenotypes. Second-generation CAR T cells incorporating CD28 domains promote effector differentiation and predominantly rely on glycolysis. Co-stimulatory domains such as 4-1BB, OX-40, and BAFF-R progressively enhance NF-κB signaling, support mitochondrial metabolism, and foster memory cell imprinting. (TCR: T-cell receptor, OXPHOS: oxidative phosphorylation, BAFF-R = B-cell activating factor receptor, Nuclear factor kappa-light-chain-enhancer of activated B-cells). Created with Biorender.com

Acknowledging the unique signaling pathways (PI3K/Akt, MAPK, NF-κB or ERK) associated with the co-stimuli CD28 and 4-1BB, each intricately connected to distinct metabolic pathways, highlights their indispensable role in both the design of a CAR construct and the ultimate determination of CAR T-cell fate (Fig. 2).

A direct comparison between CD19-CD28:ζ CARs and 4-1BB:ζ CARs shows that CD28:ζ CAR T cells have an increased glycolytic rate and a glycolytic gene signature containing genes such as GLUT1, PDK1 and SLC16A3 (monocarboxylate transporter 4 or MCT4). In contrast, 4-1BB:ζ CAR T cells are associated with more mitochondria, higher levels of OXHPOS and FAO. 4-1BB:ζ CAR T cells show elevated levels of CPT1A, which (I) is known to be a rate-limiting enzyme in the mitochondrial FAO and (II) promotes mitochondrial biogenesis [104, 108]. These findings illustrate that 28:ζ CAR T cells direct T cells towards glycolysis, whereas 4-1BB:ζ CAR T cells lean towards OXPHOS and FAO, characteristics of effector and memory T cells, respectively. When comparing both designs, CD28:ζ CAR T cells exhibit higher proportions of effector memory T cells and exhausted T cells, whereas 4-1BB promotes the formation of central memory T cells, heightened proliferation, enhanced survival, and reduced exhaustion [104]. The improved persistence of 4-1BB:ζ CARs compared to CD28:ζ T cells was also observed in multiple clinical trials [114–117]. Intriguingly, CARs targeting two antigens through split co-stimulation, providing both CD28 and 4-1BB signaling, sustain high OXPHOS while elevating glycolysis, resulting in highly metabolic CAR T cells that prove superior to single co-stimulus approaches. This highlights that mitochondrial fitness is the decisive factor in CAR T-cell functionality [118, 119].

While less common than 4-1BB and CD28, OX40 is also recognized as a costimulatory factor in CAR design. OX40 (CD134) and its ligand (OX40L) belong to the TNF(R) superfamily and are expressed across various cell types, including activated T cells. The intracellular domain of OX40 serves as a binding site for TRAF-2 and − 5 upon activation, initiating NF-κB signaling and fostering survival through an anti-apoptotic gene signature (e.g., Bcl-xl, Bcl-2) [120, 121]. Additionally, other data suggest a role for PI3K/Akt signaling upon OX40 activation [122].

Zhang et al., investigated the impact of constitutive overexpression of different costimulatory signals, parallel with second-generation 4-1BB:ζ CAR T cells. Among CD27, TIM-1, GITR and ICOS, OX40 is the most potent and can activate NF-κB and the MAPK/ERK pathway, leading to enhanced proliferation, persistence, and anti-tumor activity. However, no effects on metabolism were examined [123]. In a study by Tan et al., BCMA-targeted OX40:ζ CAR T cells were compared to 4-1BB:ζ and CD28:ζ CAR T cells. Regarding exhaustion markers, memory formation, and IFN-γ production, OX40:ζ CAR T cells outperform 4-1BB:ζ CAR T cells, which, in turn, outperform CD28:ζ CAR T cells. Gene set enrichment analysis (GSEA) analysis revealed upregulation of OXPHOS genes in OX40:ζ CAR T cells compared to 4-1BB:ζ and CD28:ζ, further underscoring the significance of the memory-mitochondrial respiration axis [124].

In a comprehensive screening study conducted by Goodman and Azimi et al., forty co-stimulatory and co-inhibitory domains were assessed within a second-generation CAR construct and compared against each other. These co-signaling domains encompass 4-1BB, CD28, CD30, CD40, TACI, BAFF-R, NTB-A, LAG-3, TIGIT, PD-1, and TIM-3. Remarkably, only three co-signaling domains, TACI, NTB-A, and B-cell activating factor receptor (BAFF-R), outperform 4-1BB and CD28 as costimulatory elements in terms of IFN-γ secretion. Both BAFF-R:ζ and TACI:ζ exhibit higher NF-κB activity compared to 4-1BB:ζ and CD28:ζ. Further analyses reveal that BAFF-R as a co-stimulus exerts the most significant impact on metabolism by reducing glycolysis and enhancing oxidative phosphorylation (OXPHOS) after repeated stimulation. Consistent with metabolic rewiring, BAFF-R:ζ CAR T cells demonstrate enhanced cytotoxicity, memory-like properties, and reduced exhaustion post-CAR stimulation [125]. BAFF has a central role in B-cell homeostasis and survival. Upon BAFF-BAFF-R interaction, TRAF3 recruitment enables NIK to activate the (non-canonical) NF-κB axis, culminating in p52/Relb translocation and the activation of survival genes and genes associated with mitochondrial biogenesis [126]. In addition to the NF-κB pathway, the PI3K-Akt axis is also activated. Interestingly, the latter induces an increase in glycolysis, which contrasts with the metabolic phenotype observed in BAFF-R:ζ CAR T cells [125, 126]. However, it can be argued that the activation of T cells might be different, using other antigen binding moieties. Hence, further investigation into the use of BAFF-R as a co-stimulatory domain in alternative CAR constructs is warranted.

## Glycolytic modulation

**Fig. 3:**
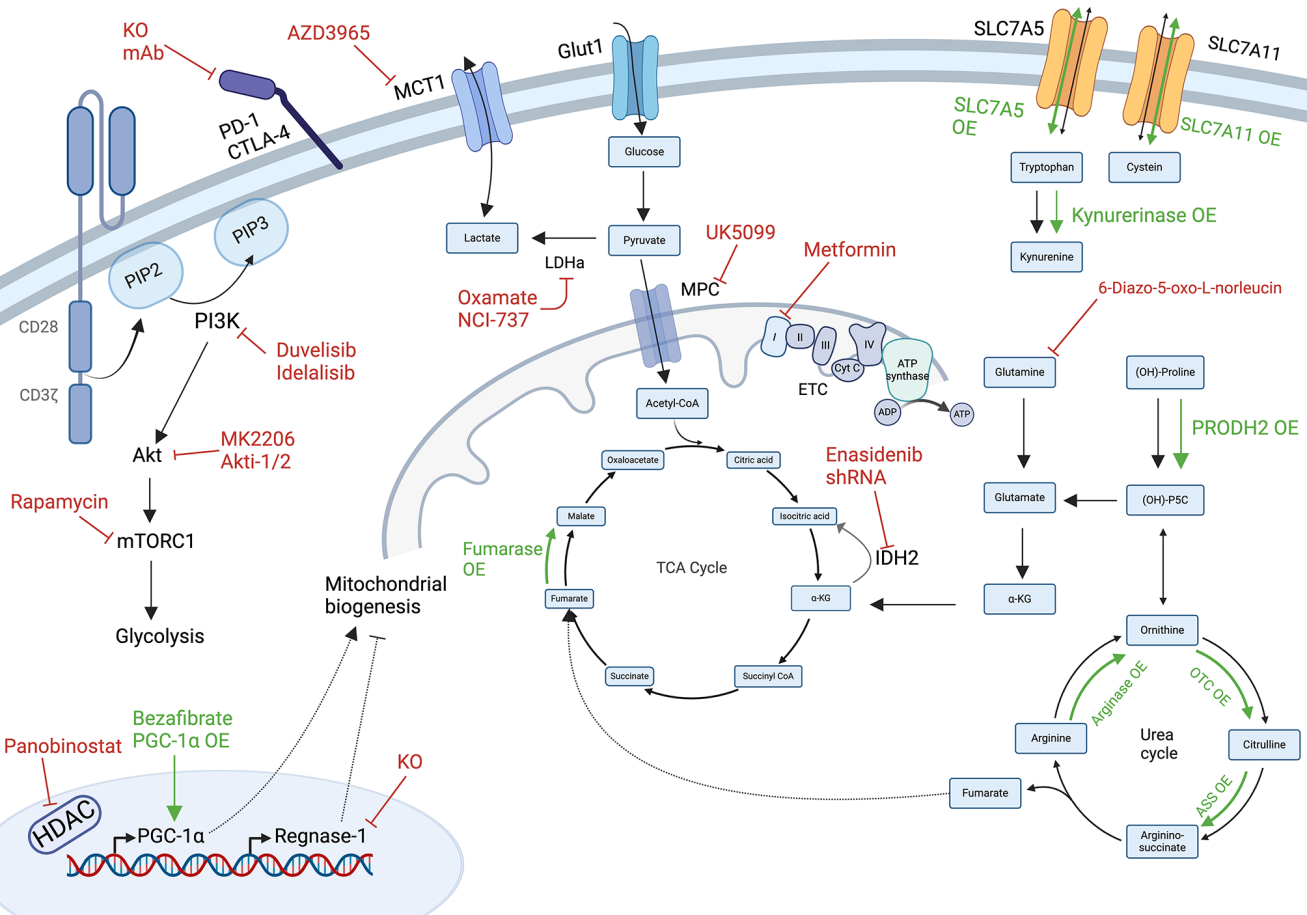
Metabolic targets to enhance CAR T-cell function and memory formation. Inhibition of targets are indicated in red, overexpression and stimulators in green. The PI3K/Akt/mTORC1 signaling pathway promotes a glycolytic gene signature, facilitating rapid energy production. Inhibition (e.g. via pretreatment of CAR T cells) of this pathway or other glycolytic proteins such as LDHA and MCTs results to increased memory formation in CAR T cells. Conversely, CAR T-cell function can be improved through several mechanisms. These include enhancing mitochondrial biogenesis via bezafibrate, overexpression of PGC-1α, or knockout of Regnase-1; preventing reductive carboxylation through IDH2 inhibition; and stimulating mitochondrial oxidation by overexpressing amino acid transporters, urea cycle enzymes, TCA cycle enzymes or inhibiting MPC. Similar beneficial effects are observed by stimulating AMPK signaling using metformin. (OE: overexpression, KO: knock-out, shRNA: short hairpin RNA, PIP2: Phosphatidylinositol(4,5)-bisphosphate, PIP3: Phosphatidylinositol(3,4,5)-trisphosphate, PI3K: Phosphatidylinositol 3-kinases, PGC-1α : Peroxisome proliferator-activated receptor-gamma coactivator 1alpha, MCT: monocarboxylate transporter, LDHA: Lactate dehydrogenase A, GLUT1: Glucose transporter 1, MPC: mitochondrial pyruvate carrier, ETC: electron transport chain, α-KG: alpha-ketoglutarate, IDH2: Isocitrate dehydrogenase 2, P5C: pyrroline-5-carboxylate, OTC: ornithine transcarbamylase, ASS: Argininosuccinate synthase or synthetase, mAb: monoclonal antibody, PD-1: programmed death-1 CTLA-4: Cytotoxic T-lymphocyte-associated protein 4, Cyt C: cytochrome C, ADP: adenosinediphosphate, ATP: adenosinetriphosphate). Created with Biorender.com

The PI3K-Akt-mTOR signaling pathway has a central role in instructing glycolysis. PI3K inhibitors have been developed as anti-tumor therapy (Fig. 3 & Table 1). Employing a combination of CAR T-cell therapy with the PI3K inhibitors LY294002 or duvelisib in patients yielded notable outcomes, including increased CAR T-cell mitochondrial fusion and respiration, and elevated levels of stem cell memory T cells – the subset exhibiting the highest self-renewal capacity [127, 128]. Duvelisib, an approved PI3K-inhibitor for chronic lymphoblastic leukemia (CLL), small lymphocytic lymphomas, and non-hodgkin lymphoma (NHL), inhibits both gamma and delta catalytic units of PI3K, and was later identified to be compromising for effector T-cell function [129, 130]. On the other hand, Idelalisib, which is also applied in CLL and follicular lymphoma treatments, selectively blocks PI3Kδ. Combining CAR T cells with Idelalisib demonstrates similar results concerning memory formation, without impairing CAR T-cell effector function [131]. Furthermore, in vitro treatment of CAR T cells with mTOR inhibitor rapamycin, Akt inhibitors MK2206 or Akt inhibitor VIII (Akti-1/2) enhances their in vivo persistence [132–134].

**Table 1:** Drugs with a link to metabolism and their effect on CAR T-cell function and fate

Drug	Target	Method	Effect on metabolism	Effect on T-cell fate	Ref.	FDA approval for
Duvelisib	PI3K	Ex vivo Pretreatment [120]	↑ Mitochondrial fusion ↑ PGC-1α	↑ Memory T cells	[128]	CLL, small lymphocytic lymphoma, NHL
Idelalisib	PI3Kδ	Ex vivo treatment	Not studied	↑ In vivo anti-tumor efficacy ↓ CD27^+^ CD28^+^ CAR T cells	[131]	CLL, follicular lymphoma
Rapamycin	mTOR	Ex vivo treatment	Not studied	↑ In vivo anti-tumor efficacy	[132]	Immunosuppressant for transplants
AKTi-1/2	Akt1, Akt2, and Akt3	Ex vivo treatment	Not studied	↑ Memory ↑ In vivo anti-tumor efficacy	[133]	Not approved
MK2206	Akt	Ex vivo treatment	Not studied	↑ CCR7 ↑ Memory subsets ↑ In vivo anti-tumor efficacy	[134]	Not approved
NCI-737	LDH	Ex vivo treatment (with or without IL-21)	↓ Glucose consumption ↓ Lactate secretion	↑ Memory ↑ In vivo anti-tumor efficacy	[100]	Not approved
UK5099	MPC	Ex vivo treatment	↓ Glucose consumption ↑ Acetyl-coA production from glutaminolysis and FAO	↑ Memory ↑ In vivo anti-tumor efficacy	[143]	Not approved
Enasidenib	IDH2	Ex vivo treatment	Glucose redirection into PPP ↑ Citrate levels	↑ Memory ↑ In vivo anti-tumor efficacy	[145]	IDH2-mutated AML and MDS
Metformin	Complex 1 of ETC	In vivo treatment	↑ AMPK	↑ Memory ↑ In vivo anti-tumor efficacy	[152]	Type 2 diabetes In clinical trials for anti-tumor potential
Panobinostat	HDAC	In vivo treatment	Not studied	↑ Memory ↑ In vivo anti-tumor efficacy	[159]	Multiple Myeloma
Bezafibrate	PPAR-α agonist	In vivo treatment	↑ Glycolysis ↑ OXPHOS ↑ FAO	↑ Effector function ↑ Memory T cells	[156]	Hypertriglyceridemia
AZD3965 AR-C155858	MCT1	In vivo treatment	↓ Glycolysis	↑ In vivo anti-tumor efficacy	[135]	AZD3965: Tested in clinical trials for DLBCL and Burkitt’s lymphoma

IL-2 is known to promote effector differentiation and aerobic glycolysis, resulting in increased lactate production [95, 100]. Using the lactate dehydrogenase A (LDHA) inhibitor NCI-737 results in a twofold increase of stem cell memory T cells and improved anti-tumor responses. The effects can be further augmented in combination with the metabolic quiescent cytokine IL-21 [100]. Other preclinical studies show that the LDHA inhibitor oxamate and the metabolite transporter monocarboxylate transporter 1 (MCT1) inhibitor AZD3965 both synergize effectively with CAR T cells [135, 136]. The described mechanisms primarily involve the inhibition of tumor-derived lactate, but they may also inhibit CAR T-cell glycolysis, thereby improving CAR T-cell function and phenotype, which warrants further investigation. Syrosingopine, an FDA approved drug for hypertension, is also a dual inhibitor of MCT1 and MCT4 [137]. In contrast to AZD3965, in vitro treatment seems to be cytotoxic for CAR T cells [135]. Oxamate, AZD3965 and syrosingopine all exhibit promising preclinical anti-MM results [137–141]. Notably, AZD3965 has already been tested in clinical trial for DLBCL and Burkitt’s lymphoma [142].

## Mitochondrial modulation

During aerobic glucose respiration, pyruvate undergoes importation into the mitochondria, is oxidated to acetyl-CoA, and subsequently metabolized in the TCA cycle. The electrons generated are transferred to the ETC for ATP production. In this section, we discuss the known targets in these processes and their effect on CAR T-cell metabolism and functioning. Although the catabolism of amino acids similarly converges on TCA intermediates, this aspect will be addressed in a separate section. This section ends with the modulation of stimulator PGC-1α and mitochondrial inhibitor Regnase-1 (Fig. 3).

The mitochondrial pyruvate carrier (MPC) transports pyruvate from the cytosol into the mitochondria, facilitating the oxidative decarboxylation to acetyl-coA. Inhibition of MPC disrupts the importation of pyruvate, leading to an increased acetyl-coA production from glutaminolysis and FAO, instead of from glucose breakdown. This metabolic rewiring induces strong H3K27 acetylation resulting in active chromatin regions for memory imprinting. While genetic perturbation of MPC in T cells results in compromised effector T cells, pre-treating CD19 CAR T cells with the MPC inhibitor UK5099 demonstrates superior CAR T-cell persistence, CD62L expression and anti-tumor activity [143].

It has been reported by Jaccard et al., that effector T cells carboxylate glutamine, thereby forming citrate from α-KG via the mitochondrial enzyme isocitrate dehydrogenase 2 (IDH2) [144]. This leads to a specific ratio of metabolites, triggering KDM5 activity, which results in demethylation of H3K4 at memory gene regions. Conversely, IDH2 inhibition disrupts this ratio, thereby increasing chromatin accessibility of memory genes encoding for CCR7, TCF1 and CD62L. CAR T cells ex vivo treated with inhibitors of IDH2 do not lose their effector function and proliferation, but are more stimulated for differentiation towards a memory phenotype [144, 145]. IDH2 acts bidirectionally, depending on the α-KG/citrate ratio. Therefore, a recent paper demonstrates that genetic perturbation of IDH2 in CAR T cells redirects glucose consumption into the pentose phosphate pathway (PPP) to provide antioxidants and rather enhances the level of citrate, thereby translocating it into the cytosol. This supports acetyl-coA-mediated activation of memory genes, by increasing histone acetylation, which again triggers a memory phenotype. Treatment of CAR T cells with the IDH2 inhibitor enasidenib has similar effects, resulting in more CD62L^+^ memory T cells, with increased survival, and less exhaustion [145]. The potential of enasidenib needs to be underscored as it is already clinically used for patients with IDH2-mutated relapsed/refractory acute myeloid leukemia (AML) [146]. Moreover, mutant IDH2 is also observed in 5% of patients with myeldysplastic syndrome (MDS) [147]. Enasidenib is EMA and FDA approved, which could facilitate its repurposing for combination therapy with CAR T-cell therapy, in AML, MDS or other malignancies.

The α-KG/citrate ratio is important to prevent demethylation and retain memory status. On the other hand, the addition of α-KG diminishes the differentiation of naïve T cells to a regulatory T-cell (Treg) phenotype by acting as co-factor for α-KGDD enzymes and promoting OXPHOS. Notably, ex vivo supplementation with α-KG reshapes the function of Treg polarized CAR T-cell towards a more pro-inflammatory state [148]. Next in the TCA cycle, α-KG is converted to succinyl-coA and succinate. Succinate involves the succinate dehydrogenase (SDH) complex, which oxidizes succinate to fumarate – a substrate for fumarase. Inhibition of either SDH or fumarase results in impaired proliferation and effector T-cell function [80, 149]. Oppositely, the genetic overexpression of fumarase strongly improves the function of CAR T-cells by decreasing fumarate levels and the succination of ZAP70 in T-cell signaling [150].

Pre-clinical studies show that metformin upregulates oxidative metabolism in CAR T cells, resulting in a long-living memory phenotypes, via upregulation of AMPK-Eomes, which suppresses PD-1 [151–153]. Pre-treating CD19 CAR T cells with metformin and rapamycin, leads to a higher mitochondrial SRC and activation of PGC-1α, essential for FAO and mitochondrial biogenesis. The combination of metformin and rapamycin promotes AMPK and inhibits mTOR, respectively, favoring a metabolic fit memory phenotype [153]. Metformin is an anti-diabetic drug, which is routinely administered in clinic, and activates AMPK by lowering ATP levels via inhibition of complex I in the mitochondrial ETC. The drug is repurposed for its detrimental effects on cancer cells. Its anti-tumor effect is being investigated in clinical trials involving both hematological and solid tumors (NCT: NCT02978547, NCT04758000, NCT03118128). Epidemiological studies already demonstrated that the use of metformin in diabetic patients with monoclonal gammopathy of undetermined significance (MGUS) is associated with a reduced risk of progression to MM and the outcome for diabetic patients with ALL [154, 155].

As mentioned earlier, mitochondrial biogenesis is regulated by PPARPGC-1α signaling. Bezafibrate acts as PPAR-α agonist and thereby elevates PGC-1α. In vivo treatment with bezafibrate enhances T-cell effector function in mice by upregulating glycolysis and OXPHOS [156]. Bezafibrate treatment results also in upregulation of both CPT1A and FAO. Although bezafibrate is not tested in a preclinical CAR T-cell context, it merits investigation. Especially since CAR T cells, genetically engineered to overexpress PGC-1α, have improved mitochondrial respiration, which leads to an increase in IFN-γ + and CCR7 + memory T cells in vivo [157]. Bezafibrate is an FDA-approved agonist, prescribed for patients with hypertriglyceridemia but also tested as cancer agent in combination with medroxyprogesterone acetate for the treatment of AML, myelodysplastic syndrome (MLS), CLL and NHL, without reported toxicities [158].

On another note, when treating CAR T cells with panobinostat, it effectively boosts their functionality through overall increases in chromatin accessibility, including memory genes such as CD62L, leading to more memory T-cell formation [159]. Although this study did not look into the effects on T-cell metabolism, it is known that panobinostat elevates PGC-1α levels, and fosters OXPHOS and FAO, all while concurrently suppressing glycolysis in malignant MM and glioma cells [160, 161]. It is worth to investigate panobinostat, since it is used for the treatment of R/R MM [162].

Furthermore, the ribonuclease Regnase-1 is identified as inhibitor of mitochondrial metabolism via the transcription factor BATF. Genetic perturbation of BATF results in decreased mitochondrial fitness, while Regnase-1 knock-out in T cells demonstrates an increased mitochondrial fitness, as reflected by the increased mitochondrial mass, volume and ΔΨM. Regnase-1 deficient CAR T cells promote TCF-1 expression, required for memory formation, resulting in long-living T cells [163–165].

## Amino acid modulation

A genome wide gain of function (GOF) CRISPR screen identified PRODH2 as booster of CAR T-cell function (Fig. 3). PRODH2 metabolizes hydroxyproline to pyrroline-3hydroxy-5-carboxylate in the catabolism of proline. GOF of PRODH2 reshapes T-cell metabolism by triggering an increased OXPHOS, and a larger number and volume of mitochondria with increased granula. PRODH2 overexpression augments effector function and improves memory phenotype following long-term co-cultures [166]. Similar effects are seen with intracellular L-arginine, which is involved in both arginine and proline metabolism [167]. T cells rely for their arginine synthesis on the low expression of argininosuccinate synthase (ASS) and ornithine transcarbamylase (OTC). Another study shows that overexpression of ASS and OTC results in increased CAR T-cell proliferation, improved in vivo persistence and less exhaustion [168]. Arginase catabolizes arginine. Overexpression of arginase or overexpression of amino acid transporters SLC7A5/SLC7A11, which upregulate arginase, positively impact mitochondrial function, resulting in improved proliferation and CAR T-cell survival in vivo [169]. Overexpression of the tryptophan transporter SLC7A5 in CAR T cells results also in more resistance to a TME, in which the amino acid availability is limited for T cells due to massive consumption by the tumor cells [169]. Kynurenine is generated in the catabolism of tryptophan and is produced by tumor cells as a so-called onco-metabolite, which affects glucose uptake by T cells. Overexpression of kynureninase in CAR T cells results in resistance to the effects of kynurenine, but also in an increased killing efficacy and memory differentiation [170]. Finally, the uptake of glutamine and its degradation during glutaminolysis is a characteristic of effector T cells. Adding the glutamine antagonist 6-Diazo-5oxo-I-norleucine in culture medium of CAR T cells results in enhanced OXPHOS, FAO, reduced in glycolysis and an increased memory T-cell phenotype [171].

All drug related interventions and genetic modifications are summarized in Tables 1 and 2, respectively.

**Table 2:** Metabolic genetic alterations in CAR T cells to improve their function and T-cell fate

Target	Genetic modification	Effect on metabolism	Effect on T-cell fate	Ref.
Fumarase	Overexpression	↓ Fumarate levels	↑ CAR T-cell function	[150]
Regnase-1	Knock out	↑ Mitochondrial mass and volume ↑ ΔΨM	↑ TCF1 expression, ↑ Memory T cells	[163–165]
PRODH2	Overexpression	↑ Number of mitochondria ↑ Mitochondria granula ↑OXPHOS	↑ Effector function ↑ Memory T cells	[166]
Argininosuccinate synthase (ASS)	Overexpression	↑ Arginine resynthesis	↑ In vivo anti-tumor efficacy ↓ Exhaustion	[168]
Ornithine transcarbamylase (OTC).	Overexpression	↑ Arginine resynthesis	↑ In vivo anti-tumor efficacy ↓ Exhaustion	[168]
SLC7A5/SLC7A11	Overexpression	↑ Amino acid uptake ↑ Intracellular arginase expression and activity	↑ CAR T-cell proliferation under low tryptophan or cystine conditions	[169]
Arginase	Overexpression	↑Arginine catabolism	↑ CAR T-cell proliferation ↑ In vivo anti-tumor efficacy	[169]
Kynureninase	Overexpression	↑ Kynunerine catabolism	↑ Killing ↑ Memory formation	[170]

## Metabolic role of PD-1 and CTLA-4 blockade

Immune checkpoint molecules serve as pivotal regulators of the immune system, acting to temper overly vigorous T-cell responses. Here, we mainly focus on PD-1 and CTLA-4, as they have been extensively studied within the context of CAR T-cell therapy.

PD-1 expression in activated or exhausted T cells regulates metabolism and represses the transcriptional regulator of mitochondrial biogenesis PGC-1α. PD-1 blockade, preventing its interaction with PD-L1, induces metabolic reprogramming of PD-1^int^ exhausted T cells, but not in PD-1^high^ T cells [68]. Studies have demonstrated that genetic disruption of PD-1 in T cells enhances anti-myeloma activity of T cells and that PD-1 knock-out in CAR T cells or combinations of nivolumab with CD19 CAR T cells are considered safe in phase 1 clinical trials [172–174]. Moreover, combining anti-PD-1 with CAR T cells also improves efficacy and outcome in clinical trials involving lymphoma patients, while also restoring BCMA CAR T-cell fitness in the treatment of MM [173–176].

Regarding CTLA-4, genetic perturbation shows promise in rescuing T cells from patients with CLL, who previously failed CAR T-cell treatment. This effect is not seen in T cells with a knock-out for both CTLA-4 and PD-1. Molecular analysis reveals that disrupting CTLA-4 enhances CD28 signaling while downregulating glycolysis in these CTLA-4 negative CAR T cells, resulting in an increased memory subset [177].

Although TIM-3, LAG-3 and TIGIT all three are associated with increased glycolysis, genetic disruption of these immune checkpoints and the implications on CAR T-cell metabolism and functioning has not studied yet [178–181].

## Conclusion

CAR T cells exhibit dynamic metabolic activity throughout their lifespan, playing a decisive role beyond mere energy provision. Metabolites actively modulate epigenetic changes at both histone and DNA levels in CAR T cells, influencing their fate decision. The metabolism of CAR T cells is also associated with the outcome of patients. CAR T cells reliant on glycolysis are deemed short-lived effectors, while those using oxidative phosphorylation and fatty acid oxidation display greater persistence and correlate with favorable long-term outcome. Metabolic modulation strategies are shown to increase the longevity of T cells. However, these pre-clinical studies mostly use T cells from healthy donors. The metabolic plasticity of T cells might be altered because of the health status, or age of the patient. Whether or not mitochondrial stimulation also can revert the pre-dysfunctional T cells in elderly patients still needs to be studied.

We present an overview of strategies that improve CAR T-cell persistence during chronic stimulation. The many genetic and pharmacological induced alterations are in the end all favoring mitochondrial respiration (OXPHOS, FAO). Not only the metabolism on its own, but also the use of TCA intermediates profoundly changes the accessibility of genes. We further highlight the potential of the FDA approved drugs used to rewire CAR T-cell’s metabolism and place them in perspective to their anti-tumor effects in hematological malignancies (Table 1). Thereby, we propose several combination strategies for clinical application in hemato-oncology, which may work on two fronts, being metabolic rewiring of CAR T cells and direct anti-tumor effects.

A potential limitation in translating metabolic compounds to the clinic lies in their off-target effects, as they may also affect the metabolism of healthy cells. Repurposing of approved drugs such as bezafibrate and enasidenib could expedite translation, given their well-established safety profiles. We feel that the effect of MCT1 or lactate dehydrogenase inhibitors should be further investigated, as these compounds can work on multiple levels; possibly rewiring of CAR T cells towards a favorable metabolism, targeting the Warburg effect in malignant cells, and modulating the tumor micro-environment, since there will be a decrease in the immunosuppressive metabolite lactate.

Another strategy relies on genetic engineering of CAR T cells to alter metabolic pathways. First of all the design of CAR T cells profoundly impacts their metabolism and functionality. Extracellular antigen-binding scFvs may self-aggregate, leading to unwanted signaling and metabolic reprogramming, whereas nanobodies offer a solution to this issue. In addition, the selection of co-stimulatory domains significantly influences T-cell metabolism and fate. It is worth to explore alternative co-stimuli beyond the conventional 4-1BB and CD28 domains. Especially the co-stimulatory domain of BAFF-R deserves recognition as co-stimulus and should be further studied in a clinical setting, ideally in a nanoCAR.

Finally, since T cells are already isolated for the insertion of the CAR, an additional DNA sequence can be easily added to the transgene. However, safety concerns such as malignant transformation should be further investigated. Alternatively, safety can be increased by including a suicide gene, however this further impacts the size of the transgene [182].

Altogether, in the popular and evolving field of T-cell metabolism, it is worthy to further explore the potential of metabolic rewiring as a promising approach to enhance CAR T-cell therapy.

## Data Availability

No datasets were generated or analysed during the current study.

## Abbreviations

α-KGDD: α-KG-dependent dioxygenases
2-HG: 2-hydroxyglutarate
ACC2: Acetyl-CoA carboxylase 2
Acetyl-coA: Acetyl-coenzyme A
AMPK: AMP-activated protein kinase
BCMA: B-cell maturation antigen
CAR: Chimeric antigen receptor
CPT1A: Carnitine palmitoyl transferase 1 A
DLBCL: Diffuse large B-cell lymphoma
Cilta-cel: Ciltacabtagene autoleucel (cilta-cel)
DNMT: DNA methyltransferase
DRP1: Dynamin-related protein 1
ER: Endoplasmic reticulum
ETC: Electron transport chain
GSEA: Gene set enrichment analysis
HAT: Histone acetyltransferase
HDAC: Histone deacetylase
Ide-cel: Idecabtagene vicleucel
IDH2: Isocitrate dehydrogenase 2
KDM: Lysine demethylase
LDHA: Lactate dehydrogenase A
R/R: Relapsed and/or refractory
MCT1: Monocarboxylate transporter 1
MGUS: Monoclonal Gammopathy of Unknown Significance
MM: Multiple Myeloma
MPC: Mitochondrial pyruvate carrier
Opa-1: Optic athrophy-1
OXPHOS: Oxidative phosphorylation
FAO: Fatty acid oxidation
TSCM: Stem cell memory T cells
TCM: Central memory T cells
TEm: Effector memory T cells
Treg: Regulatory T-cell
PBMC: Peripheral blood mononuclear cells
PGC-1α: Peroxisome proliferator-activated receptor gamma coactivator 1-alpha
PHDs: Prolyl hydroxylases
SAM: S-adenosyl-methionine
SIRT: Sirtuins
SRC: Spare respiratory capacity
TCA: Tricarboxylic acid
TET: Ten-eleven translocation
TNF(R): Tumor necrosis factor (receptor)
ΔΨM: Mitochondrial membrane potential
